# Prognostic value of neutrophil-to-lymphocyte ratio, lactate dehydrogenase, D-dimer, and computed tomography score in patients with coronavirus disease 2019

**DOI:** 10.18632/aging.203501

**Published:** 2021-09-08

**Authors:** Yu-Qing Cai, Xiao-Bin Zhang, Hui-Qing Zeng, Xiao-Jie Wei, Lan Hu, Zhen-Yu Zhang, Quan Ming, Qiu-Ping Peng, Li-Da Chen

**Affiliations:** 1 Department of Clinical Medicine, Fujian Medical University, Fujian, China; 2 Department of Pulmonary and Critical Care Medicine, Zhongshan Hospital, Xiamen University, Teaching Hospital of Fujian Medical University, Fujian, China; 3 Department of Pulmonary and Critical Care Medicine, Third People's Hospital Affiliated to Fujian University of Traditional Chinese Medicine, Fujian, China; 4 Department of Gastroenterology, Optic Valley Division of Tongji Hospital, Tongji Medical College, Huazhong University of Science and Technology, Wuhan, China; 5 Department of Geriatrics, Zhongshan Hospital, Xiamen University, Teaching Hospital of Fujian Medical University, Fujian, China; 6 Yichang Third People's Hospital, Third People's Hospital Affiliated to Sanxia University, Hubei, China; 7 Department of Pulmonary and Critical Care Medicine, Zhangzhou Affiliated Hospital of Fujian Medical University, Fujian, China

**Keywords:** COVID-19, neutrophil-to-lymphocyte ratio, lactate dehydrogenase, D-dimer, CT score

## Abstract

Background: This study aimed to explore the significance of neutrophil-to-lymphocyte ratio (NLR), lactate dehydrogenase (LDH), D-dimer, and CT score in evaluating the severity and prognosis of coronavirus disease 2019 (COVID-19).

Methods: Patients with laboratory-confirmed COVID-19 were retrospectively enrolled. The baseline data, laboratory findings, chest computed tomography (CT) results evaluated by CT score on admission, and clinical outcomes were collected and compared. Logistic regression was used to assess the independent relationship between the baseline level of the four indicators (NLR, LDH, D-dimer, and CT score) and the severity of COVID-19.

Results: Among the 432 patients, 125 (28.94%) and 307 (71.06%) were placed in the severe and non-severe groups, respectively. As per the multivariate logistic regression, high levels of NLR and LDH were independent predictors of severe COVID-19 (OR=2.163; 95% CI=1.162-4.026; *p=*0.015 for NLR>3.82; OR=2.298; 95% CI=1.327-3.979; *p=*0.003 for LDH>246 U/L). Combined NLR>3.82 and LDH>246 U/L increased the sensitivity of diagnosis in patients with severe disease (NLR>3.82 [50.40%] vs. combined diagnosis [72.80%]; *p*=0.0007; LDH>246 [59.2%] vs. combined diagnosis [72.80%]; *p<*0.0001).

Conclusions: High levels of serum NLR and LDH have potential value in the early identification of patients with severe COVID-19. Moreover, the combination of LDH and NLR can improve the sensitivity of diagnosis.

## INTRODUCTION

Since December 2019, coronavirus disease-2019 (COVID-19), caused by a novel coronavirus (SARS-CoV-2), has rapidly spread worldwide, causing a major public health issue [1]. COVID-19 is obviously a huge challenge for the global healthcare system [2], with the mortality of patients being related to the healthcare burden [3]. Therefore, a reasonable distribution of medical resources is particularly important. In turn, early identification of critical patients is crucial for the rational allocation of resources and the improvement of patient prognosis.

According to reports, hematological changes are more prominent in patients with severe COVID-19 than in patients with non-severe disease [4]. The neutrophil-to-lymphocyte ratio (NLR), lactate dehydrogenase (LDH), and D-dimer are closely associated with the poor prognosis of COVID-19 [5, 6]. Without other clinical parameters, computed tomography (CT) evaluation is an independent prognostic factor in patients with COVID-19 [7]. However, there are few data comparing these four indicators. Therefore, in this study, we aimed to compare the prediction efficiency of NLR, LDH, D-dimer, and CT scores and evaluate the significance of the optimum cutoff. Subsequently, a combined diagnosis analysis was also performed to evaluate whether the combination of these indicators could improve diagnosis efficiency.

## RESULTS

### Baseline, laboratory and imaging characteristics

In this retrospective study, a total of 432 patients with COVID-19 were enrolled, including 202 (47%) females and 230 (53%) males with an average age of 52.88 years. Fever (308, 71.3%), cough (270, 62.5%), expectoration (130, 30.1%), and fatigue (128, 29.6%) were the most common symptoms. Hypertension (92, 21.3%) was the most common comorbidity.

The patients have been divided into the severe (125/432, 28.94%) and non-severe (307/432, 71.06%) groups based on disease severity. In terms of the baseline characteristics, patients in the severe group had a more advanced average age than those in the non-severe group (59.60±16.65 years vs. 50.14±16.26 years, *p*<0.0001). The severe group also had a higher incidence of comorbidities, such as hypertension (*p*<0.0001), diabetes (*p*<0.0001), and chronic obstructive pulmonary disease (*p*=0.009). As for the clinical laboratory findings, lower levels of lymphocytes (*p*<0.0001) and higher levels of white blood cells (*p*=0.023), neutrophils (*p*<0.0001), C-reaction proteins (*p*<0.0001), LDH (*p*<0.0001), D-dimer (*p*<0.0001), and NLR (*p*<0.0001) were detected in the severe group than in the non-severe group. Regarding the CT results, 96.0% (120/125) of patients in the severe group had bilateral lung involvement, 32% (40/125) had consolidation, and 3.2% (4/125) had pleural effusion. A significant difference in terms of CT score was also observed between the two groups (6 [4–9] for the severe group vs. 6 [4–7] for the non-severe group, *p*<0.0001) (Table 1).

**Table 1:** Baseline, laboratory and imaging characteristics.

**Variable**	**Total (n=432)**	**Severe group (n=125)**	**Non-severe group (n=307)**	***p* value**
Age(years)	52.88±16.91	59.60±16.65	50.14±16.26	<0.0001
Gender				0.072
Female-n (%)	202(47)	50(40)	152(49.5)	
Male-n (%)	230(53)	75(60)	155(50.5)	
Clinical symptom-n (%)				
Fever	308(71.3)	94(75.2)	214(69.7)	0.252
Fatigue	128(29.6)	46(36.8)	82(26.7)	0.037
Dyspnea	35(8.1)	20(16)	15(4.9)	< 0.0001
Pharyngalgia	34(7.9)	12(9.6)	22(7.2)	0.394
Cough	270(62.5)	84(67.2)	186(60.6)	0.198
Chest tightness	47(10.9)	22(17.6)	25(8.1)	0.004
Diarrhea	20(4.6)	6(4.8)	14(4.6)	0.914
Myalgia	46(10.6)	19(15.2)	27(8.8)	0.05
Expectoration	130(30.1)	35(28)	95(30.9)	0.545
Headache	19(4.4)	6(4.8)	13(4.2)	0.795
Poor appetite	53(12.3)	19(15.2)	34(11.1)	0.236
Comorbidities-n (%)				
Hypertension	92(21.3)	53(42.4)	39(12.7)	<0.0001
Diabetes	56(13)	31(24.8)	25(8.1)	<0.0001
COPD	25(5.8)	13(10.4)	12(3.9)	0.009
Renal insufficiency	9(2.1)	8(6.4)	1(0.3)	<0.0001
Cardiac insufficiency	8(1.9)	7(5.6)	1(0.3)	0.01
Hepatic insufficiency	30(6.9)	16(12.8)	14(4.6)	0.002
Anemia	13(3.0)	7(5.6)	6(2.0)	0.089
Clinical laboratory				
White blood cell-10^^9^/L	5.25±2.52	5.76±3.19	5.04±2.16	0.023
Lymphocyte-10^^9^/L	1.28±0.62	1.04±0.70	1.37±0.55	<0.0001
Neutrophil-10^^9^/L (IQR)	2.98(2.11-4.18)	3.41(2.32-5.50)	2.82(2.08-3.77)	<0.0001
CRP-mg/L(IQR)	22.32(9.15-37.7)	45.2(14.85-55.8)	22.32(7.7-22.32)	<0.0001
Platelet-10^^9^/L	173.02±80.88	163.03±83.74	177.08±79.47	0.102
D-dimer-μg/ml (IQR)	0.55(0.44-0.82)	0.62(0.50-1.42)	0.52(0.42-0.68)	<0.0001
LDH-U/L(IQR)	210(170-267.75)	265(207.5-356)	196(162-235)	<0.0001
NLR (IQR)	2.33(1.51-3.94)	3.84(2.06-7.13)	2.03(1.41-3.25)	<0.0001
CT manifestations				
CT score (IQR)	6(4-7.75)	6(4-9)	6(4-7)	<0.0001
Bilateral lung involved-n (%)	359(83.1)	120(96)	239(77.9)	<0.0001
Ground glass opacity-n (%)	426(98.6)	124(99.2)	302(98.4)	0.830
Consolidation-n (%)	96(22.2)	40(32)	56(18.2)	0.002
Pleural effusion-n (%)	5(1.2)	4(3.2)	1(0.3)	0.042
Pleural thickening-n (%)	5(1.2)	1(0.8)	4(1.3)	1.000

Abbreviation: COPD, chronic obstructive pulmonary disease; CRP, C-reactive protein; NLR, neutrophil - to- lymphocyte ratio; LDH, lactate dehydrogenase.

### Predictive value of NLR, LDH, D-dimer, and CT score

**Figure 1:**
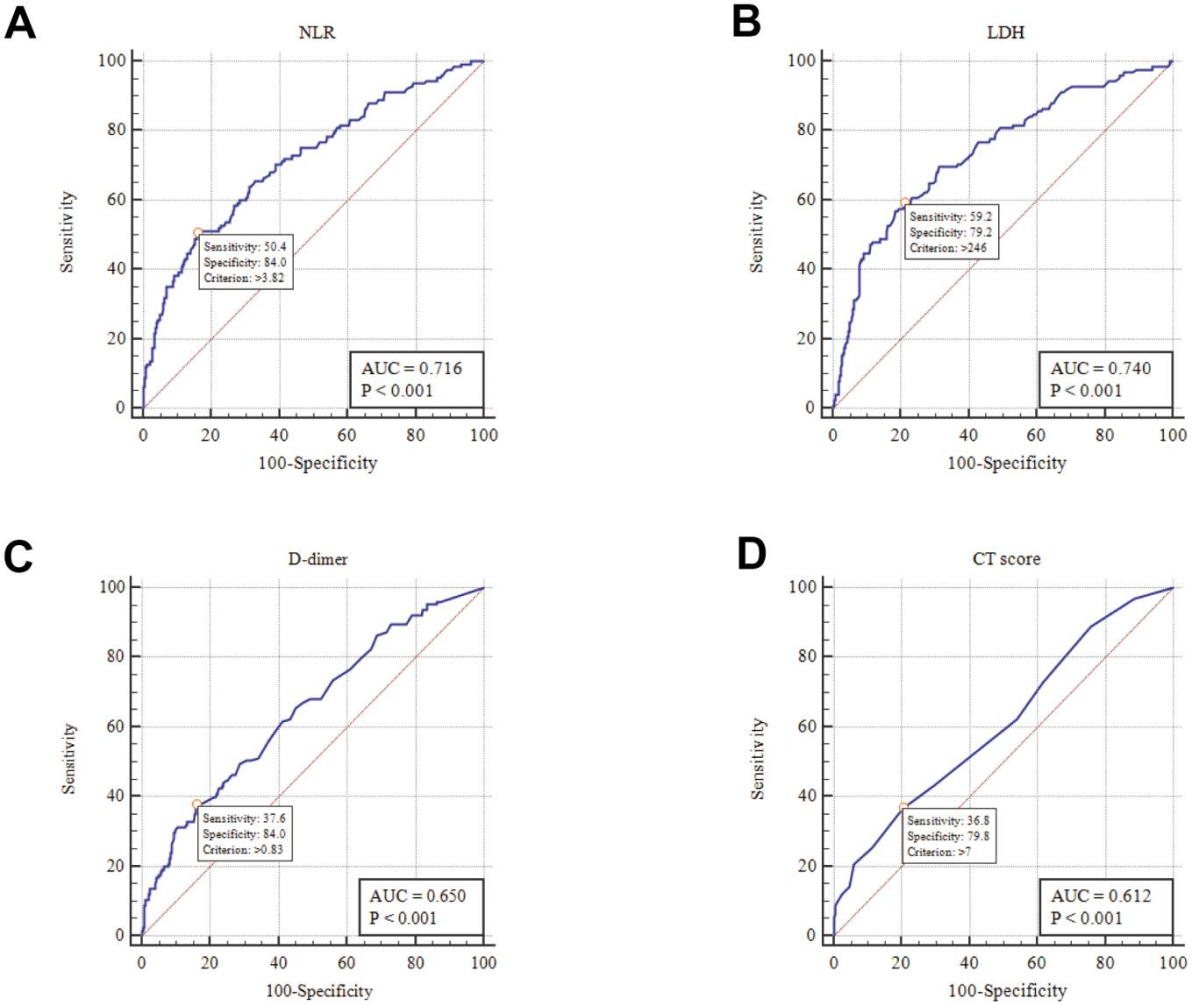
ROC analysis of NLR, LDH, D-dimer and CT score in disease risk prediction (**A**) NLR; (**B**) LDH; (**C**) D-dimer; (**D**) CT score.

As shown in Table 1, NLR, LDH, D-dimer, and CT scores were significantly higher in the severe group than in the non-severe group. Based on the receiver operating characteristic (ROC) curve, the area under the curve (AUC) was 0.716 for NLR, 0.740 for LDH, 0.650 for D-dimer, and 0.612 for CT score, indicating a certain diagnostic value for the severity of disease (Figure 1 and Table 2). In addition, the optimum cutoff values from the ROC were 3.82, 246 U/L, 0.83 μg/mL, and 7 for NLR, LDH, D-dimer, and CT score, respectively (Table 2).

**Table 2:** Area under ROC curve and optimum cutoff.

**Variables**	**Assessment of validity**								
	**AUC**	**Optimum cutoff**	**Sensitivity**	**Specificity**	**Predictive value**			**Likelihood ratio**	
					**Positive**	**Negative**		**Positive**	**Negative**
NLR	0.716	3.82	50.4%	84.04%	56.3%	80.6%		3.16	0.59
LDH(U/L)	0.740	246	59.2%	79.15%	53.6%	82.7%		2.84	0.52
D-dimer(μg/ml)	0.650	0.83	37.6%	84.04%	49%	76.8%		2.36	0.74
CT-score	0.612	7	36.8%	79.8%	42.6%	75.6%		1.82	0.79

Abbreviation: ROC, receiver operator characteristic curve; NLR, neutrophil-to-lymphocyte ratio; LDH, lactate dehydrogenase.

We assumed that when the levels of NLR, LDH, D-dimer, and CT score on admission exceeded the optimum cutoff, the patients were prone to develop severe or critical disease types. Patients were then divided into different subgroups according to the optimum cutoff.

As Table 3 shows, 25.9% (112/432), 31.9% (138/432), 22.2% (96/432), and 25% (108/432) of patients had high levels of NLR, LDH, D-dimer, and CT score on admission, respectively. After grouping, the distribution of baseline NLR [63/125 (50.4%) vs. 49/307 (16%); *p*<0.0001], LDH [74/125 (59.2%) vs. 64/307 (20.8%); *p*<0.0001], D-dimer [47/125 (37.6%) vs. 49/307 (16%); *p*<0.0001], and CT score [46/125 (36.8%) vs. 62/307 (20.2%); *p*<0.0001] over the optimum cutoff in the two groups were significant (Table 3).

**Table 3:** Baseline after grouping.

	**Total n=432**	**Severe group n=125**	**Non -severe group n=307**	***p* value**
NLR				
>3.82	112(25.9%)	63(50.4%)	49(16%)	*p*<0.0001
≤3.82	320(74.1%)	62(49.6%)	258(84%)	
LDH (U/L)				
>246	138(31.9%)	74(59.2%)	64(20.8%)	*p*<0.0001
≤246	294(68.1%)	51(40.8%)	243(79.2%)	
D-dimer(μg/ml)				
>0.83	96(22.2%)	47(37.6%)	49(16%)	*p*<0.0001
≤0.83	336(77.8%)	78(62.4%)	258(84%)	
CT score				
>7	108(25%)	46(36.8%)	62(20.2%)	*p*<0.0001
≤7	324(75%)	79(63.2%)	245(79.8%)	

Abbreviation: ROC, receiver operator characteristic curve; NLR, neutrophil-to-lymphocyte ratio; LDH, lactate dehydrogenase.

Univariate analysis indicated that high levels of NLR, LDH, D-dimer, and CT score were positively correlated with disease severity (OR=5.350; 95% CI=3.361-8.518; *p<*0.0001 for NLR; OR=5.509; 95% CI=3.511-8.646; *p<*0.0001 for LDH; OR=3.173; 95% CI=1.976-5.094; *p<*0.0001 for D-dimer; OR=2.301; 95% CI=1.455-3.638; *p<*0.0001 for CT score). After adjusting for other statistically significant indices, the predictive value of NLR>3.82, LDH>246 U/L persisted (OR=2.163; 95% CI=1.162-4.026; *p=*0.015 for NLR; OR=2.298; 95% CI=1.327-3.979; *p=*0.003 for LDH). By contrast, the relationship among D-dimer>0.83 μg/mL, CT score>7, and disease severity weakened (OR=1.209; 95% CI=0.626-2.334; *p=*0.571 for D-dimer; OR=1.519; 95% CI=0.71-3.247; *p=*0.281 for CT score). In addition, fatigue (OR=1.978; 95% CI=1.127-3.473; *p=*0.018), chest tightness (OR=2.265; 95% CI= 1.011-5.074; *p=*0.047), hypertension (OR=2.534, 95% CI=1.259-5.099; *p=*0.009), C-reactive protein (OR=1.013; 95% CI= 1.003-1.023; *p=*0.011), and bilateral lung involvement (OR=3.890; 95% CI=1.356-11.154; *p=*0.011) were still positively correlated with disease severity (Table 4).

**Table 4:** The univariate and multivariable logistic regression.

**Variables**	**Unadjusted odds ratio (95%CI)**	***p* value**	**Adjusted odds ratio (95%CI)**	***p* value**
NLR	5.350(3.361 - 8.518)	<0.0001	2.163(1.162 - 4.026)	0.015
LDH(U/L)	5.509(3.511 - 8.646)	<0.0001	2.298(1.327 - 3.979)	0.003
D-dimer(μg/ml)	3.173(1.976 - 5.094)	<0.0001	1.209(0.626 - 2.334)	0.571
CT score	2.301(1.455 - 3.638)	<0.0001	1.519(0.71 - 3.247)	0.281
Age	1.036(1.022 - 1.050)	<0.0001	0.994(0.975 - 1.014)	0.561
Fatigue	1.598(1.026-2.488)	0.038	1.978(1.127-3.473)	0.018
Dyspnea	3.708(1.831 - 7.509)	<0.0001	1.348(0.507-3.585)	0.55
Chest tightness	2.409(1.302 - 4.460)	0.005	2.265(1.011-5.074)	0.047
Hypertension	5.058(3.103 - 8.245)	<0.0001	2.534(1.259 - 5.099)	0.009
Diabetes	3.720(2.091 - 6.619)	<0.0001	1.304(0.597 -2.848)	0.506
COPD	2.853(1.264 - 6.441)	0.012	1.019(0.314 -3.303)	0.975
Renal insufficiency	20.923(2.589 -169.118)	0.004	4.788(0.449 -51.025)	0.195
Cardiac insufficiency	18.153(2.210 -149.133)	0.007	2.245(0.135 -37.251)	0.573
Hepatic insufficiency	3.072(1.451 - 6.505)	0.003	2.209(0.842 -5.792)	0.107
CRP (mg/L)	1.025(1.017 - 1.033)	<0.0001	1.013(1.003 -1.023)	0.011
Bilateral lung involved	6.828(2.683 - 17.381)	<0.0001	3.890(1.356 -11.154)	0.011
Consolidation	2.109(1.312 - 3.390)	0.002	1.303(0.6 - 2.829)	0.504
Pleural effusion	10.116(1.119 -91.421)	0.039	5.097(0.409 -63.513)	0.206

Abbreviation: NLR, neutrophil - to- lymphocyte ratio; LDH, lactate dehydrogenase; COPD, chronic obstructive pulmonary disease; CRP, C-reactive protein.

### Evaluation of the multi-parameter model

According to logistic regression, NLR>3.82 and LDH>246 U/L were statistically significant risk factors (Table 4). As shown in Table 2, the sensitivity of NLR>3.82 and LDH>246 U/L in predicting the severity of COVID-19 were 50.40% and 59.20%, respectively. Further evaluation was performed to judge whether the combined diagnosis model of the two indices can improve prediction sensitivity.

Table 5 indicates that the combined diagnosis of NLR>3.82 and LDH>246 U/L could increase the sensitivity of predicting disease severity [NLR>3.82 (50.40%) vs. combined diagnosis model (72.80%); *p* =0.0007; LDH>246 (59.2%) vs. combined diagnosis model (72.80%); *p<*0.0001].

**Table 5:** Comparison of univariate and combined diagnosis model.

**Variables**	**Sensitivity**	**Specificity**	***p* value**
NLR>3.82	50.40%	84.04%	0.0007^1^
LDH>246U/L	59.20%	79.15%	<0.0001^2^
Combined diagnosis model	72.80%	69.71%	

Abbreviation: NLR, neutrophil - to- lymphocyte ratio; LDH, lactate dehydrogenase; combined diagnosis model, NLR>3.82 and LDH>246U/L; 0.0007^1^, *p* value between NLR>3.82 and combined diagnosis model; <0.0001^2^, *p* value between LDH>246U/L and combined diagnosis model.

## DISCUSSION

A total of 432 patients with COVID-19 were included in this retrospective study. In the univariate analysis, we found that high levels of NLR, LDH, D-dimer, and CT score were significantly correlated with COVID-19 severity. After adjusting for other statistically significant indices, the predictive value of NLR>3.82 and LDH>246 U/L persisted. This indicates that when NLR exceeded the cutoff point, the risk of serious disease increased by 2.163 times. Moreover, the risk of LDH over the optimum cutoff increased by 2.298 times. By contrast, the value of D-dimer>0.83 μg/mL and CT score>7 in predicting disease severity was weak and these indices could therefore not be recommended as independent predictors. In addition, the risk of severity was closely related to fatigue, chest tightness, hypertension, and C-reactive protein. Furthermore, combining NLR>3.82 and LDH>246 U/L can improve the sensitivity of disease risk prediction.

Immune dysfunction plays an important role in the severity of COVID-19 [8]. Recent studies have elucidated that neutropenia and lower lymphopenia can be observed in patients with severe COVID-19 [9, 10]. The NLR simultaneously considers the lymphocytes and neutrophils, and several studies have shown the predictive value of NLR in distinguishing patients with severe and critical COVID-19. In a study of the dynamic changes in lymphocyte subsets and cytokine profiles in patients with COVID-19, NLR was found to be a prognostic factor for the early identification of severe cases [11]. A cohort of patients with COVID-19 also proved that, after adjustment for confounding factors, the risk of in-hospital mortality increased by 8% for each unit increase in NLR [12]. Another study conducted by Yang et al. [5] in 93 patients with COVID-19 demonstrated that NLR can be used as an independent indicator for poor clinical outcome, and that the largest AUC for NLR was 0.841, with 63.6% specificity and 88% sensitivity. However, the outcome requires further evaluation because of limited sample diversity. The predictive value of NLR in the present study was consistent with the abovementioned studies. Moreover, the sample size and diversity in the present study were improved by collecting data from two clinical centers, which strengthens the reliability of our conclusions. We found that the optimum cutoff for NLR was 3.82, and the AUC was 0.716. Moreover, the sensitivity and specificity of NLR>3.82 were 50.40% and 84.04%, respectively. Moreover, as per the multivariate logistic regression, NLR>3.82 can be used as an independent predictor for disease risk (OR=2.163; 95% CI=1.162-4.026; *p=*0.015).

Elevation of LDH is one of the most common laboratory abnormalities in patients with COVID-19. Acute lung injury is highly associated with LDH [13]. A systematic literature review and meta-analysis showed that LDH levels >245 U/L can predict the progression of COVID-19 [6]. In a study of the risk factors for death in cancer patients with COVID-19, elevated LDH levels were closely related to increased mortality [14]. Furthermore, in another retrospective analysis of 120 patients with COVID-19, the patients with severe disease had higher LDH levels than patients with mild disease (mean 200.8 U/L for mild vs. mean 342.8 U/L for severe) [15]. The predictive value of LDH was further confirmed in our study. Our ROC analysis showed that the AUC for LDH was 0.74, and that the optimum cutoff was 246 U/L. The sensitivity and specificity were 59.2% and 79.15%, respectively. Logistic regression indicated that the risk of serious disease increased by 2.298 times when LDH was above the optimum cutoff (OR=2.298; 95% CI=1.327-3.979; *p=*0.003). In addition, the sensitivity of disease risk prediction can be improved by combining LDH >246 U/L with NLR>3.82. (NLR>3.82 [50.40%] vs. combined diagnosis model [72.80%]; *p*=0.0007; LDH>246 [59.2%] vs. combined diagnosis model [72.80%]; *p<*0.0001). However, the specificity was decreased (NLR>3.82 [84.04%] vs. combined diagnosis model [69.71%]; *p*=0.0007; LDH>246 [79.15%] vs. combined diagnosis model [69.71%]; *p<*0.0001).

Furthermore, the sensitivity, specificity, and AUC for NLR and LDH were not sufficiently high. Due to the different admission times of patients with COVID-19 and the acute aggravation of some patients after admission, the value of admission indicators may have been underestimated. However, compared with other studies [5, 11, 16], the sample size and diversity of patients with COVID-19 have increased the reliability of the results in this study. More importantly, the optimum cutoff can indicate the risk of acute aggravation in patients with COVID-19 in the present study. Furthermore, our study provides more evidence for the establishment of a multiparameter diagnosis model. The combination of indicators increases the possibility of disease progression. And the role of primary screening in emergency needs to be further confirmed.

Coagulation disorders are more common in patients with severe disease than in patients with mild disease [17, 18]. A study conducted by Zhang et al. [19] showed that a D-dimer level ≥2.0 μg/mL (four-fold increase) could effectively predict the mortality of patients with COVID-19. In our study, after balancing the confounding factors, the logistic regression showed that D-dimer >0.83 μg/mL could not be used as an independent predictor of disease risk (OR=1.209; 95% CI=0.626-2.334; *p=*0.571). In a dynamic study of hematological parameters in patients with COVID-19, the D-dimer level was higher in the severe group than in the non-severe group on days 1, 7, and 14 (*p*<0.05) [20]. This suggests that due to different admission times, the ability of D-dimer to predict disease risk may be weakened. In addition to the prognostic value of D-dimer in patients with COVID-19, the predictive value of D-dimer might be affected by other factors, such as hormone therapy and antibiotic therapy. Because the baseline level of D-dimer varies greatly in patients, the value of D-dimer dynamic monitoring may be higher in patients with COVID-19 [21]. Nevertheless, further research is required to evaluate the significance of D-dimer in evaluating the severity of COVID-19.

Patients with COVID-19 have lung involvements with imaging changes [22, 23]. In different stages of the disease, the CT manifestations are different, which are important for the diagnosis and staging of patients [24]. Using the same semi-quantitative scoring system, a multi-center paired cohort study conducted by Liu et al. [25] showed that CT changes are obvious during the acute exacerbation of COVID-19, accompanied by an increase in CT score. This indicates that an elevated CT score may predict a poor outcome. Another retrospective single-center study indicated that the CT score had a high diagnostic value in patients with severe COVID-19. ROC analysis showed that the AUC for the CT score was 0.918. The optimum cutoff CT score was 7.5. The sensitivity and specificity were 82.6% and 100%, respectively [8]. However, the study only analyzed imaging without combining it with clinical data. Significant differences in the number of patients between the severe-critical and non-severe groups also affected the accuracy of the results. In the present study, after combining the clinical data, the CT score cannot be used as an independent predictor of disease risk (OR=1.519; 95% CI=0.71-3.247; *p=*0.281). A study by Zhang B et al. [26] demonstrated that the severity of lung abnormalities evaluated by CT score might be associated with laboratory parameters. Therefore, due to the correlation between CT score and laboratory parameters, the ability to independently predict the disease risk from CT scores may be attenuated. Additional investigations are warranted to assess whether CT score can be an independent predictor of disease risk.

This study has some limitations. First, owing to the different disease severities among the patients, as well as the different medical resources available, the time from onset to admission might not be representative, which could have affected the level of the four parameters considered on admission. Moreover, the representativeness of the CT score and D-dimer may have also been affected by the different admission times. Second, other clinical data and test results were not included in the analysis, which may have caused bias, weakening the reliability of the results. Third, it should be noted that the CT score was a subjective semi-quantitative evaluation method, to a certain degree. In the future research, it is necessary to conduct dynamic research on indicators and combine more indicators to meet different clinical needs.

## CONCLUSIONS

As independent factors, the serum levels of NLR and LDH were significantly correlated with COVID-19 severity. Therefore, we recommend NLR and LDH as predictors for evaluating the severity of COVID-19.

## MATERIALS AND METHODS

### Study design and participants

From January 20, 2020, to March 30, 2020, a total of 432 patients confirmed COVID-19 by the laboratory in designated treatment hospitals (Optic Valley division of Tongji Hospital, Tongji Medical College, Huazhong University of Science and Technology, Wuhan and Yichang Third People's Hospital, Hubei Province) were enrolled_._ The patients were divided into 2 groups based on the seventh edition of the New Coronavirus Pneumonia Diagnosis and Treatment Program published by the Chinese National Health Commission [27]: the mild and moderate types were classified as non-severe group and the severe and critical were included into severe group. The disease is classified as severe if one of the following items is met: 1) shortness of breath, respiratory rate ≥ 30 beats per min; 2) the oxygen saturation ≤ 93% in a resting state; 3) arterial partial pressure of oxygen (PaO_2_) / concentration of oxygen (FiO_2_)≤ 300 mmHg (1 mmHg = 0.133 kPa); 4) pulmonary images show that the lesions progressed more than 50% within 24-48h. The critical should meet one of the following conditions: 1) respiratory failure and need mechanical ventilation; 2) shock; and 3) other organ failures need ICU monitoring and treatment.

### Date collection

The data of patients’ demographic characteristics, comorbidities, laboratory findings, chest computed tomography (CT) results, and clinical outcomes were extracted from electronic medical records. The BC 3000 auto hematology analyzer (Mindray Medical International, Inc., Shenzhen, China) was used for routine blood tests of hospitalized patients. Biochemical and inflammatory markers were obtained on a Beckman Coulter AU5800 (Beckman Coulter Co, Brea, CA, USA). CT image acquisition and scoring A thoracic CT scan was performed before or after 2 days of admission in all patients. According to the extent of involvement of each lobe, each lobe was scored as 0 (0%), 1 (1-25%), 2 (26-50%), 3 (51-75%), or 4 (76-100%). The total severity score (TSS) is the cumulative score of five lobes (score range 0-20) [8, 28]. In order to ensure the accuracy of the data, all data were checked by two physicians, respectively.

### Statistical analysis

According to the different data distribution, continuous variables were described as mean ± standard or median (Inter-quartile range, IQR), and groups were compared by student’s t-test or Mann-Whitney U test based on the data distribution. Categorical variables were presented as n (%) and analyzed by Pearson’s chi-square. Receiver operator characteristic (ROC) was used to evaluate the efficacy of NLR, LDH, D-dimer and CT score and get the optimum cutoff. Logistic regression was used to access the predictive value for disease risk. The statistical software needed is SPSS version 21 and Medcalc (version 19.1). A value of *p*<0.05 was considered statistically significant.

### Ethics approval and consent to participate

The study was approved by the Ethics Committee of Zhongshan Hospital, Xiamen University and Second affiliated Hospital of Fujian Medical University.

### Availability of data and material

All data generated or analyzed during this study are included in this published article.
